# Tuberculosis induced autoimmune haemolytic anaemia: a systematic review to find out common clinical presentations, investigation findings and the treatment options

**DOI:** 10.1186/s13223-018-0236-y

**Published:** 2018-03-26

**Authors:** Devarajan Rathish, Sisira Siribaddana

**Affiliations:** 1 Department of Pharmacology, Faculty of Medicine and Allied Sciences, Rajarata University of Sri Lanka, Saliyapura, 50008 Sri Lanka; 2 Department of Medicine, Faculty of Medicine and Allied Sciences, Rajarata University of Sri Lanka, Saliyapura, 50008 Sri Lanka

**Keywords:** Tuberculosis, Autoimmune haemolytic anaemia, Antituberculosis medicines, Case reports, Direct coombs test

## Abstract

**Background:**

Tuberculosis induced autoimmune haemolytic anaemia is a rare entity. The aim of this study was to explore its common presentations, investigation findings and treatment options through a systematic review of published reports.

**Methods:**

PubMed, Trip, Google Scholar, Science Direct, Cochrane Library, Open-Grey, Grey literature report and the reference lists of the selected articles were searched for case reports in English on tuberculosis induced auto-immune haemolytic anaemia. PRISMA statement was used for systematic review. Quality assessment of the selected reports was done using the CARE guidelines.

**Results:**

Twenty-one articles out of 135 search results were included. Thirty-three percent of patients were reported from India. More than half had fever and pallor. The mean haemoglobin was 5.77 g/dl (SD 2.2). Positive direct coombs test was seen in all patients. Pulmonary tuberculosis (43%) was most prevalent. Twenty-nine percent of patients needed a combination of anti-tuberculosis medicines, blood transfusion and steroids. Higher percentage of disseminated TB induced AIHA (67%) needed steroids in comparison to the other types of TB induced AIHA (13%).

**Conclusions:**

Rarer complications of tuberculosis such as auto-immune haemolytic anaemia should be looked for especially in disease-endemic areas. Blood transfusion and steroids are additional treatment options along with the anti-tuberculosis medicines.

**Electronic supplementary material:**

The online version of this article (10.1186/s13223-018-0236-y) contains supplementary material, which is available to authorized users.

## Background

Tuberculosis (TB) infection is caused by *Mycobacterium tuberculosis.* Pathogenesis of TB includes primary, latent and reactivation TB [1]. Pulmonary TB is the most frequent type [2], however, it can affect almost all the human organs. Immuno-compromised individuals are more susceptible. Globally TB is among the most common causes of mortality. The majority of the deaths occur in developing countries and India tops the list. Asia and Africa recorded 61% and 26% of new TB cases respectively in 2015 [2].

TB affects the CVS, CNS, gastrointestinal, genito-urinary and haematological systems [3]. Blood disorders due to tuberculosis are rare. However, TB can affect all three-cell lines in the blood. Anaemia, leukopaenia, leucocytosis, basophilia, monocytosis, thrombocytopenia and pancytopaenia are reported haematological complications of TB. Autoimmune haemolytic anaemia (AIHA) is an extremely rare occurrence in TB [4, 5]. Incidence of AIHA is 1–3 per 100,000 population per year [6]. The pathogenesis of TB induced haemolysis is unclear but, altered immune response is implicated [7].

AIHA is due to increased red cell destruction by autoantibodies and the direct coombs test is positive [1]. Warm type AIHA occurs at ≥ 37 °C, mediated by IgG and the cold type occurs at < 37 °C, involving IgM antibodies [1]. Corticosteroid is the first line in treating warm type followed by splenectomy, which usually are ineffective in the cold type. Blood transfusion may be necessary for both types. Commonly a lymphoproliferative disorder causes warm type while, *Mycoplasma* and viral infections commonly cause cold type. Finding and treating the underlying cause of AIHA is essential [1]. Infection caused by *Mycobacterium tuberculosis* may lead to an immune response that results in the production of IgG, IgM or both antibodies. This would lead to the mediation of warm, cold or mixed type of AIHA.

This study aimed at systematically reviewing globally reported cases of tuberculosis induced AIHA concerning its presentation, investigations and treatment. The objective was to synthesize knowledge related to this rare clinical entity. In 2014, a review on TB induced AIHA cases was reported. This review adds new cases and an in-depth analysis for the previous review which had 16 cases in it [8].

## Methods

The review was conducted based on the Preferred Reporting Items for Systematic review and Meta-Analysis (PRISMA) statement [9].

### Eligibility criteria

All published case reports on tuberculosis induced AIHA were included. No studies were excluded based on the year of publication or patient population. Reports published in languages other than English were excluded.

### Information sources and Search strategy

**Fig. 1:**
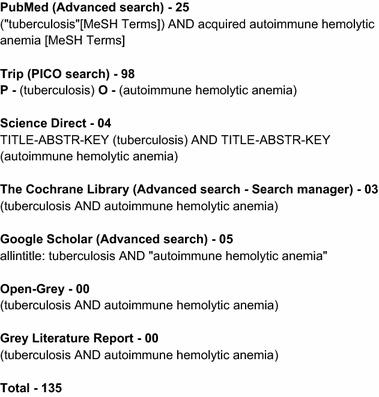
Keywords for databases and the number of search results

The search was done on the 31st of January 2017. Electronic databases and grey literature were searched using strings of keywords (Fig. 1). Following databases were used for the search: PubMed (Advanced search) [10], Trip (PICO search) [11], Google Scholar (Advanced search) [12], Science Direct (Expert search) [13], Cochrane Library (Advanced search—Search manager) [14], Open-Grey [15] and Grey literature report [16]. In-addition, the reference lists of the selected studies were checked for relevant articles. MeSH and other relevant terms related to each search engine were used to produce a maximum yield.

### Study selection

DR independently screened the titles and abstracts of all identified studies according to the eligibility criteria. The full-text article was examined when abstracts were unclear. SS independently reviewed the selected studies to confirm the eligibility.

### Data collection process, data items and data analysis

Year of publication, demographic details, common clinical presentations, investigation findings and the treatment options were extracted from the selected study. Data for the following variables were sought: population—patients with tuberculosis, outcome—autoimmune haemolytic anaemia and study design—case report. All units of measurements were presented in SI units. The CARE guidelines which were “designed to increase the accuracy, transparency, and usefulness of case reports” was used to assess the quality of the case reports [17]. A score of one was given for each item outlined in the CARE guidelines with a maximum score of 29 for a case report. Data were analysed using Microsoft Excel. Descriptive statistics were used to describe data.

## Results

### Selected case reports

**Fig. 2:**
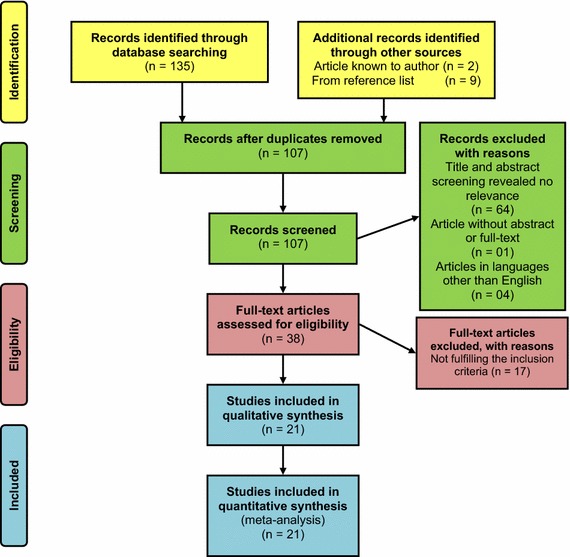
Flow diagram showing the selection process of articles for this review, according to PRISMA 2009

A total of 135 results were found from the databases (Fig. 1). Further, nine articles were selected from their reference lists. Two articles known to authors were also included [18, 19]. After removal of duplicates, 107 articles were included for the title and abstract screening. Out of which 64 articles were excluded due to irrelevance to the study objective. One article was excluded due to unavailability of abstract or full-text [20], and another 04 articles were excluded as they were in French [21], Turkish [22], Spanish [23] and Russian [24] languages. The full-texts of the remaining 38 articles were examined and 17 were excluded, as they did not fulfil the selection criteria of the review. Following the above screening steps, 21 articles [18, 19, 25–43] were selected for the review (Fig. 2). Out of the selected case reports eight were found from PubMed, two from Google Scholar, nine from screening references of available case reports and two known to authors. According to the quality assessment, the mean score achieved by the selected articles was 23.24 (SD ± 1.92). The maximum score was 27 out of 29 and the minimum score was 20. Consent from patient to publish was not reported in any of the 21 case reports.

### Demographic data

Additional file 2 contains data from each of the 21 reports selected for this review. Eight case reports were from South-East Asia (38%), four from Western Pacific (19%), four from Europe (19%), three from the region of Americas (14%) and two from Eastern Mediterranean (10%). There were no reports from the African region. Country wise, India topped the list with seven reported cases followed by Taiwan (02) and France (02). All other countries (Brazil, Canada, China, Kuwait, Saudi Arabia, Sri Lanka, Tunisia, Turkey, United Kingdom, and the United States of America) reported one case each. According to this review, the first case reported on TB induced AIHA was by Cameron in 1974 from Scotland, United Kingdom [43]. The first case reported from South-East Asia was from Sri Lanka by Siribaddana and Wijesundera in 1997 [18]. The latest report was by Kolla et al. in 2016 from India [19].

The mean age of the patients was 28.38 (SD 14), with an age range of 8–58. Three cases were less than 18 years, and were from India [35, 36] and Tunisia [33]. There were 12 (57%) female patients. All patients, except three, belonged to the nations from where their illness was reported. The three patients, reported from Kuwait [26], France [34] and Saudi Arabia [37], originated from Ethiopia, Morocco and Indonesia respectively.

### Clinical presentations

Out of 21 patients, chronic cough was present in 48% of the patients, of which 30% (3/10) was productive. Haemoptysis was seen in one patient reported from India in 2016 [19]. Fever (> 37 °C) was present in 81%, out of which 47% (8/17) had an intermittent type. Night sweats were seen in two (10%). Loss of appetite was seen in 14.3% and weight loss in 38%. Forty-three percent presented within a month, 29% within one to 3 months and the rest presented after 3 months of onset of their symptoms.

### Predisposing factors to acquire TB

Past history of pulmonary TB was seen in patients from India [27] and Scotland [43]. The patient reported from Canada in 1975 [24] had a past history of tuberculous salpingitis. Both parents of an Indian patient reported history of TB [36]. Another Indian [35] had a contact history for pulmonary TB via his uncle who was treated for 6 months prior to the patients presentation. In-addition, the Moroccan patient reported from France [34] had travelled to her native country 7 months before the presentation. No other significant predisposing factors were noted from the rest of the 15 patients.

### Examination findings

On examination, pallor was seen in 62% of the patients. The majority (57%) did not show a lymphadenopathy. Axillary and cervical lymphadenopathies, which were seen in 33% (3/9) patients, were the most frequent. Abnormal lung signs were seen only in 34% of the patients with the crackles being the most common finding (100% - 7/7) Clinically splenomegaly was noted in 19% (4/21) and hepatomegaly in 10% (2/21).

### Laboratory investigation

The mean haemoglobin was 5.77 g/dl (SD 2.2, range 1.8–10.3) (Table 1). Electrophoresis was done in eight patients out of which 37.5% (3/8) had polyclonal-gammopathy and one had haemoglobin SC (from America in 1978) [41]. Tuberculin skin test was reported in 11 patients. Only five had the width of the induration reported. The mean size of induration was 19.2 mm (SD 17.2, range 10–30). The rest were reported as non-reactive (n = 3), positive (n = 2) and strongly positive (n = 1). Sputum staining for acid-fast bacilli was done in ten patients and 60% (6/10) were positive. Sputum culture for *Mycobacterium tuberculosis* was done in ten patients and 70% (7/10) were positive. All patients were positive for direct coombs test. Indirect coombs test was done in five patients and 60% (3/5) were positive. Out of the 15 cases that classified AIHA, 46.7% were warm, 40%—cold and 13.3%—mixed. 14 patients were reported with a blood film result. Polychromasia (8/14) and spherocytosis (7/14) was seen among more than half of whom had a blood film (Table 2). Bone marrow was carried out in 15 patients and 67% (10/15) of them had erythroid hyperplasia. Anti-nuclear antibody was performed in 71% (15/21) of patients out of which only two were positive. Rheumatoid factor was tested in only two patients and both were within normal limits.

**Table 1:** Summary of results of laboratory investigations

Name of investigation (Unit)	Cases reported (%)	Normal range	Range	Mean (SD)
Haemoglobin (g/dl)	21 (100) (M:F = 9:12)	M: 14–18 F: 12–16	M:3.5–10.3 F:1.8–9.3	M: 5.41 (2.1) F: 6.03 (2.3)
MCV (fl/red cell)	11 (52.4)	7.8–11	74.3–128.9	96.94 (15.6)
Reticulocytes (%)	17 (81)	0.3–2.3	0.06–21.4	10.9 (6.1)
LDH (U/l)	15 (71.4)	110–240	455–1902	909.4 (431.3)
Total Bilirubin (µmol/l)	14 (66.7)	5.1–20.5	13.7–82.1	52.2 (21)
Direct Bilirubin (µmol/l)	12 (57)	0.0–6.8	1.7–51.3	18.6 (15.3)
Haptoglobin (g/l)	08 (38)	0.36–1.95	0–0.38	0.14 (0.14)
ESR (mm/hr)	07 (33.3) (M:F = 2:5)	M: 0–15 F: 0–20	M:42–120 F:30–150	M: 81 (55.2) F: 85 (47.7)

*SD* standard deviation, *Min* minimum, *Max* maximum, *M* male, *F* female, *MCV* mean corpuscular volume, *LDH* lactate dehydrogenase, *ESR* erythrocyte sedimentation rate

**Table 2:** Findings of blood films reported from the 14 cases

Findings	Frequency out of 14 (%)
Polychromasia	08 (57)
Spherocytosis	07 (50)
Anisopoikilocytosis	05 (36)
Anisocytosis	03 (21)
Macrocytosis	03 (21)
Normoblasts	03 (21)
Red cell agglutinins	02 (14)
Target cells	02 (14)
Elliptocytosis	01 (07)
Macrocytic normochromic red cells	01 (07)
Microcytic hypochromic red cells	01 (07)
Normochromic cells	01 (07)
Poikilocytosis	01 (07)
Tear drop cells	01 (07)

### Radiological findings

Seventeen patients have had a chest x-ray out of which 18% (3/17) was reported as normal, the rest had findings suggestive of TB (Additional file 1). An ultrasound scan was reported in six cases and 50% (3/6) of them showed splenomegaly. Seven patients had computed tomography of the chest; lymphadenopathies were seen in 71% (5/7) and tree-in-bud pattern in 26% (2/7). Bronchiectasis was reported only from Tunisia [33]. High-resolution computed tomography was performed in two and both revealed upper lobar features suggestive of TB.

### Site of TB infection

Lung (43%) was the most common site of TB, followed by disseminated TB (29%), lymph node TB (14%), abdominal TB (9%) and genitourinary TB (5%).

### Treatment modalities and survival

Anti-tuberculosis medicines were given to all 21 patients. Blood transfusion was needed in 48% of the patients. However, steroids were given for 38%. It was impossible to summarize the doses of steroid used due to the variation in reporting. The decision on steroid therapy was based on the benefit vs. risk assessment between treatment of AIHA and the risk of worsening of TB. In-addition, if the patient’s haemolytic anaemia failed to respond to anti-TB medicines and/or blood transfusion some physicians have opted for steroid therapy. The combination of anti-tuberculosis medicines, blood transfusion and steroids was needed in 29% (6/21). Out of the patients with warm type, only one 14% (1/7) needed steroids [26], whereas it was 33% in cold (2/6) and 100% in mixed (2/2). Half of the patients who received steroids for AIHA had disseminated TB, whereas only 15% had disseminated TB in the non-steroid group. In other words, 67% of patients with disseminated TB induced AIHA, needed steroid therapy. Only 13% of patients with other types of TB induced AIHA, needed steroid therapy. No adverse effects were reported with steroid use. All survived, except the one patient reported from India in 2013 [29].

### Cases from India

One-third of cases (34%) were reported from India. They were reported from Mangalore, 2016 [19], Jaipur, 2015 [25], Bangalore, 2013 [27], Nagpur, 2013 [29], Chennai, 2011 [31], Varanasi, 2005 [35] and New Delhi, 2004 [36]. Considering the highest prevalence of TB infection and the highest reporting of TB induced AIHA cases, it is useful to summarize the clinical and laboratory findings from India.

Mean age was 19.6 (SD 9), with 57% being females. Fever, chronic cough, haemoptysis, night sweats, loss of appetite and weight loss were present in 86, 43, 14, 00, 29 and 14% respectively. Pallor, lymphadenopathy, crepitation, splenomegaly and hepatomegaly were noted in 86, 43, 29, 43 and 29% respectively. Mean haemoglobin was 4.86 g/dl (SD 2.4). 72% were unclassified for AIHA type. 14% each were classified as cold and mix types. There were no Indian patients with warm type. Anisopoikilocytosis (57%) was the most common finding in blood films. 71% of patients had erythroid hyperplasia in bone marrow. Lung was the most common site of tuberculosis infection (43%). The combination of blood transfusion and steroids was needed in 29%. All survived, except one from Nagpur, 2013 [29]. PRISMA 2009 checklist is available as Additional file 2.

## Discussion

Anaemia alone or in combination with other blood abnormalities was seen in 63% of miliary TB patients [44]. Proposed mechanisms were nutritional deficiencies, malabsorption due to GIT tuberculosis, associated anorexia and immune mediated marrow suppression [7]. TB induced AIHA is a rare entity in a patient with anaemia and TB. AIHA is treated with steroids, and in the presence of TB, it would result in adverse consequences [7, 8]. However, such consequences were not observed in any of the cases reviewed. Routine investigations confirmed the diagnosis of TB and AIHA, among the cases of this review.

Pallor and hepatosplenomegaly were uncommon in TB and provide diagnostic clue for associated AIHA. Most had pulmonary TB with good response to anti-TB treatment. All patients had a positive direct coomb’s test [18, 19, 25–43]. The decision to perform a coomb’s test was based on the physician’s clinical assessment. Blood transfusion and steroids were needed for 48 and 38% of the patients respectively. Higher percentage of patients with disseminated TB induced AIHA (67%) needed steroid therapy in comparison to those who had other types of TB induced AIHA (13%). Contrary to usual management of AIHA only 14% of the warm type required steroids, whereas it was 33% for the cold type.

In concordance with the global prevalence of TB [2], the incidence of TB-induced AIHA was high in South-east Asia and India. Having no cases reported from Africa was surprising. However, an Ethiopia and a Moroccan were reported to have TB induced AIHA from Kuwait [26] and France [34] respectively.

The mean quality assessment score for the selected articles were 23.24 (SD 1.92) out of a total of 29 per article. This shows an acceptable level of quality of the selected articles. Differences in reporting of examination findings, laboratory results and treatment doses were noted. Even differences in confirmation of TB infection were noted. Strict adherence to a case reporting guide like the CARE guidelines [17] would have minimized it. These limited the review in producing a comprehensive summary of TB induced AIHA. Nevertheless, the review has produced optimum information on TB induced AIHA. Future prospective studies on TB induced AIHA are methodologically challenging considering the rare nature of the disease. However, continuous reporting of similar cases will help improve the understanding of this rare entity.

## Conclusions

Tuberculosis induced auto-immune-haemolytic-anaemia should be looked for, in patients presenting with pallor, lymphadenopathy and hepatosplenomegaly along with other symptoms of tuberculosis. This is important, especially in tuberculosis-endemic regions. Treatment with anti-tuberculosis medicines with steroids should be offered for such patients.

## Additional files

**Additional file 1.** Data sheet of the review on TB induced AIHA, 2017. This provides the data extracted for the review.

**Additional file 2.** PRISMA 2009 checklist. This provides the PRISMA 2009 checklist related to this systematic review.

## Abbreviations

AIHA: autoimmune haemolytic anaemia
CARE: case reports
CNS: central nervous system
CVS: cardiovascular system
ESR: erythrocyte sedimentation rate
F: female
GIT: gastro-intestinal tract
LDH: lactate dehydrogenase
M: male
Max: maximum
Min: minimum
PICO: population, intervention, comparison and outcome
PRISMA: preferred reporting items for systematic reviews and meta-analyses
SD: standard deviation
TB: tuberculosis
WHO: World Health Organization

## References

[1] Kumar P, Clark M (2017). Clinical Medicine.

[2] WHO. Tuberculosis. Geneva: World Health Organization; 2017. http://www.who.int/mediacentre/factsheets/fs104/en/. Accessed 26 Mar 2017.

[3] Shah M, Reed C (2014). Complications of tuberculosis. Curr Opin Infect Dis.

[4] Coburn RJ, England JM, Samson DM, Walford DM, Blowers R, Chanarin I (1973). Tuberculosis and blood disorders. Br J Haematol.

[5] Glasser RM, Walker RI, Herion JC, Hill C (1970). The significance of hematologic abnormalities in patients with tuberculosis. Arch Intern Med.

[6] Liebman HA, Weitz IC (2017). Autoimmune hemolytic anemia. Med Clin North Am.

[7] Cameron SJ. Tuberculosis and the blood–a special relationship?. Tubercle. 1974;55:55–72. http://www.ncbi.nlm.nih.gov/pubmed/4534375. 10.1016/0041-3879(74)90067-1 4534375

[8] Bahbahani H, Al-Rashed M, Almahmeed M. Tuberculosis and autoimmune hemolytic anemia: Case report and literature review. J Appl Hematol. 2014;5:164. http://www.jahjournal.org/text.asp?2014/5/4/164/146953.

[9] Moher D, Liberati A, Tetzlaff J, Altman DG, Prisma Group (2009). Preferred reporting items for systematic reviews and meta-analyses: the PRISMA statement. PLoS Med.

[10] PubMed. NCBI. 2017. https://www.ncbi.nlm.nih.gov/pubmed. Accessed 26 Mar 2017.

[11] Trip Database. 2017. https://www.tripdatabase.com/. Accessed 26 Mar 2017.

[12] Google Scholar. 2017. https://scholar.google.com/. Accessed 26 Mar 2017.

[13] Science Direct. 2017. http://www.sciencedirect.com/. Accessed 26 Mar 2017.

[14] Cochrane Library. 2017. http://www.cochranelibrary.com/. Accessed 26 Mar 2017.

[15] OpenGrey. 2017. http://www.opengrey.eu/. Accessed 26 Mar 2017.

[16] Grey Literature Database. New York Acad Med. 2017. http://www.greylit.org/. Accessed 26 Mar 2017.

[17] CARE case report guidelines. 2017. http://www.care-statement.org/. Accessed 26 Mar 2017.

[18] Siribaddana SH, Wijesundera A (1997). Autoimmune haemolytic anaemia responding to anti-tuberculous treatment. Trop Doct.

[19] Kolla G, Acharya V, Balanthimogru P, Mani A, Ruman S (2016). Rare offshoot of a common malady anaemia and tuberculosis. J Clin Diagn Res.

[20] Kim SW, Choi SW, Cho BK, Houh W, Lee JW (1995). Tuberculosis cutis orificialis: an association with Evans’ syndrome. Acta dermato-venereologica.

[21] Bouldouyre MA, de Reviers O, Boussadia A, Gros H, Delassus JL, Bakir R (1983). Healing of autoimmune hemolytic anemia only with anti-tuberculosis treatment. Press Méd Paris Fr.

[22] Fakültesi T, Hematoloji Ç, Dal B, Dal PA, Dal B (2007). Hodgkin lenfoma, tüberküloz ve otoimmün hemolitik anemili olgu : Olgu sunumu. Türk Onkoloji Dergisi.

[23] Ortega M, Zambrano I, Martinez C, Rabadan F, Fajardo J, Mora JA (1990). Miliary tuberculosis associated with cold agglutinin anemia in a patient with a history of chronic malaria. An Med Interna.

[24] Gorbatovskaia NS, Rassokhin VM (1975). Acquired autoimmune hemolytic anemia with an isolated splenic tuberculosis. Sov Med.

[25] Anurag L, Richa C (2015). Cold agglutinin induced hemolytic anemia in a patient with pulmonary tuberculosis. Int J Med Res Health Sci.

[26] Bahbahani H, Al-Rashed M, Almahmeed M (2014). Tuberculosis and autoimmune hemolytic anemia: case report and literature review. J Appl Hematol.

[27] Kumar KS, Prabhudas VM, Bheemaraya D, Chaitra CS (2013). Autoimmune hemolytic anemia in a patient with endo-bronchial tuberculosis. J Evol Med Dent Sci.

[28] Safe IP, O’brien C, Ferreira FRL, de Souza MLVD, Ramasawmy R (2013). Tuberculosis associated with transient hemolytic anemia responsive to tuberculosis chemotherapy: a case report. Braz J Infect Dis.

[29] Somalwar A, Aher A, Zanwar V, Kolpakwar K (2013). Tubercular lymphadenitis and abdominal tuberculosis with autoimmune hemolytic anemia and severe intravascular hemolysis. Ann Trop Med Public Health.

[30] Wu B, Rong R (2012). Cold agglutinin syndrome with severe haemolytic anaemia in a patient diagnosed of disseminated tuberculosis and concomitant *Mycoplasma pneumoniae* infection. Transfus Med.

[31] Nandennavar M, Cyriac S, Krishnakumar TG (2011). Immune hemolytic anemia in a patient with tuberculous lymphadenitis. J Glob Infect Dis.

[32] Chen H-C, Huang L-T, Wang H-C (2010). Severe hemolytic anemia due to active pulmonary tuberculosis. Fooyin J Health Sci.

[33] Khemiri M, Zouari S, Barsaoui S (2008). Autoimmune bicytopenia in pulmonary tuberculosis. Report of a pediatric case. Respir Med CME.

[34] Morell S, Lambert M, Queyrel V, Launay D, Quemeneur T, Hachulla E (2006). Tubercular adenitis and Evans’ syndrome. Lupus.

[35] Gupta V, Bhatia BD (2005). Abdominal tuberculosis with autoimmune hemolytic anemia. Indian J Pediatr.

[36] Bakhshi S, Rao IS, Jain V, Arya LS (2004). Autoimmune hemolytic anemia complicating disseminated childhood tuberculosis. Indian J Pediatr.

[37] Abdullah AA, Mohammed AL, Fahad MAM (2002). Autoimmune hemolytic anemia associated with intestinal tuberculosis. Ann Saudi Med.

[38] Turgut Mehmet, Uzun Oðuz, Kelkýtlý Engin, Özer Okay (2002). Pulmonary tuberculosis associated with autoimmune hemolytic anemia: an unusual presentation. Turk J Haematol.

[39] Kuo PH, Yang PC, Kuo SS, Luh KT (2001). Severe immune hemolytic anemia in disseminated tuberculosis with response to antituberculosis therapy. Chest.

[40] Blanche P, Rigolet A, Massault PP, Bouscary D, Dreyfus F, Sicard D (2000). Autoimmune haemolytic anaemia revealing miliary tuberculosis. J Infect.

[41] Murray HW (1978). Transient autoimmune hemolytic anemia and pulmonary tuberculosis. N Engl J Med.

[42] Semchyshyn S, Cecutti A (1975). Abdominal pregnancy complicated by genital and renal tuberculosts and hemolytic anemia. Fertil Steril.

[43] Cameron SJ (1974). Tuberculosis and the blood—a special relationship?. Tubercle.

[44] Glasser RM, Walker RI, Herion JC. The significance of hematologic abnormalities in patients with tuberculosis. Arch Intern Med. 1970;125:691–5. http://www.ncbi.nlm.nih.gov/pubmed/5437894. 5437894

